# The Score Takes Care of Itself

**DOI:** 10.1308/rcsann.2023.0113

**Published:** 2024-01-01

**Authors:** B Rogers

**Figure:**
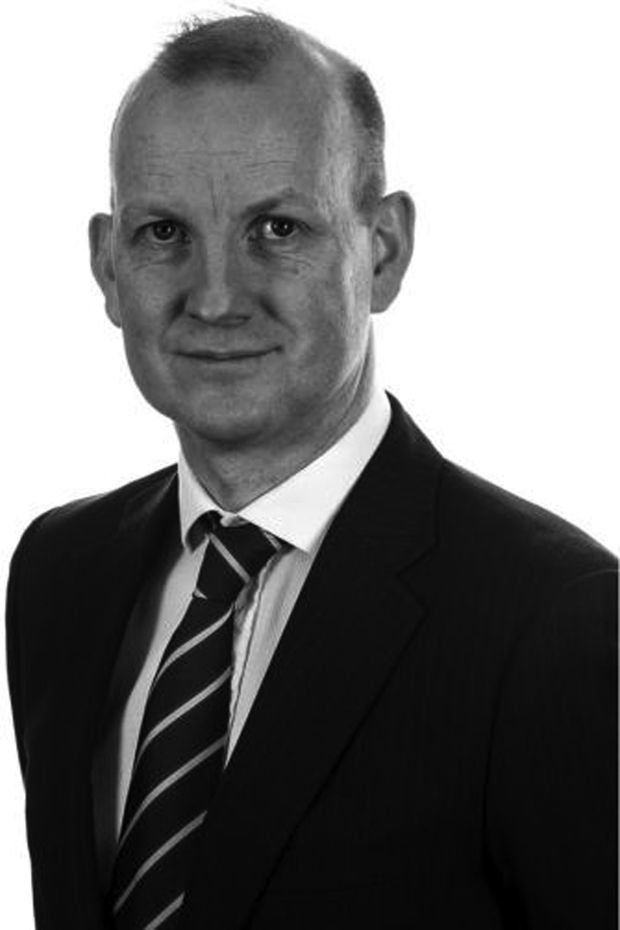
**Benedict Rogers,** Editor-in-Chief *Annals* of the Royal College of Surgeons of England

With the conclusion of 2023, the surgical profession continues to address numerous challenges, including inefficiencies of service, training difficulties, carbon footprint, the role of artificial intelligence and clinician burnout. All these issues are complex, multi-faceted, and evolving with no simple solutions, and they demand ongoing research and analysis. I am glad that the *Annals* now attracts a growing caseload of research on these ‘non-clinical’ topics that contributes to the body of evidence.

Some may argue that ‘non-clinical’ topics such as those outlined above carry less academic weight or importance. As Editor-in-Chief*,* I disagree and consider such topics of equal value as pure clinical papers. Furthermore, as a pan-surgical journal, the *Annals* affords the optimal vehicle to publish and promote topics that more specialised journals would have difficulty accepting within their terms of reference. This is a significant strength of our journal, and we will continue to encourage authors to submit work on these topics in addition to traditional clinical research.

Notwithstanding the above, and while the breadth of topics considered by our journal has increased since 1947, the rigour of peer review continues, and the methodological strength of any submitted research needs to be optimal. The journal welcomes well-planned projects with clearly stated aims and power analysis to afford statistical clarity. Opportunistic, retrospective post-hoc studies are rarely of a standard or value the wider readership expects and gains from.

This ‘wider readership’ will likely become even wider, as this January 2024 edition marks the first fully gold open access issue for the *Annals* – providing immediate free access at first publication. This is a significant evolution for the journal that I am pleased to highlight. Open access facilitates faster and broader dissemination of research through a variety of digital media. The *Annals* will continue to publish high-quality surgical research that is relevant to the wider surgical profession and incorporates a wide breadth of topics. Specifics about our open access policy are available on the journal website.^1^

It is wholly unjust for me to accept any credit for the transition to open access, a process that has taken a year to complete. As with any significant change, the process has incorporated proposals, presentations, votes, meetings and wider discussions. Subsequently, implementing the changes on various digital platforms and amending agreements with publishers and online depositories has involved much time. The professional and dedicated work of Mandy Webb (Head of Publishing, RCS England) and Morgane Tixier (Publishing Coordinator, RCS England) are the principal reasons the journal can now evolve to open access. I am indebted to both.

I am optimistic that 2024 will see the *Annals* continue to progress due to the wide and relevant research it promotes, the academic rigour of the peer review process and the faster availability of published work. The core of the journal, the standards it maintains, continue to be the wide body of reviewers. Reviews of depth and insight dictate the standard of articles the readers see, and most articles are forged into greater manuscripts through the comments and thoughts of the reviewers. In this edition, we publish a list, with great thanks, of colleagues who provide a quiet yet valued service to the *Annals*.

We exist in a data-driven world – clinically, academically, financially and through publication media. Numerous metrics are now available for journals, for example, usage, citations, altmetrics, impact factor and subscription rates. Bill Walsh, the respected coach of the San Francisco 49ers American football team, famously titled his book “*The Score Takes Care of Itself*”^2^ which I would recommend. Furthermore, I strive to ensure the *Annals* continues to focus on the people (authors, reviewers, editors, publishing team) and the process (peer review and publication) and let the metrics take care of themselves.
